# Delamanid and bedaquiline resistance patterns in *Mycobacterium tuberculosis* in Iran: A cross-sectional analysis

**DOI:** 10.1016/j.nmni.2024.101437

**Published:** 2024-05-26

**Authors:** AmirHossein Akbari Aghababa, Mohammad Javad Nasiri, Parviz Pakzad, Elnaz Sadat Mirsamadi

**Affiliations:** a Department of Microbiology, Faculty of Biological Sciences, North Tehran Branch, Islamic Azad University, Tehran, Iran; b Department of Microbiology, School of Medicine, Shahid Beheshti University of Medical Sciences, Tehran, Iran; c Department of Microbiology, Faculty of Medicine, Tehran Medical Sciences, Islamic Azad University, Tehran, Iran

**Keywords:** *Mycobacterium tuberculosis*, Antimicrobial resistance, Bedaquiline, Delamanid, Multidrug-resistance

## Abstract

**Introduction:**

The surge of multidrug-resistant TB (MDR-TB) in Iran poses a significant challenge to global healthcare. The introduction of delamanid (DLM) and bedaquiline (BDQ), two potent antimycobacterial drugs, marks a crucial advance. Nevertheless, as resistance in *Mycobacterium tuberculosis* is on the rise in Iran and resistance to these newer medications is emerging, investigations in this field are of utmost importance.

**Methods:**

In this cross-sectional study, 38 MDR-TB strains were collected from five distinct regional TB laboratories in Iran. The clinical isolates were confirmed as *M. tuberculosis* using the phenotypic tests and IS6110-based PCR assay. Drug susceptibility testing (DST) for isoniazid, rifampicin, ethambutol, DLM, and BDQ was performed using WHO-approved methods. Sequencing was used to investigate genetic mutations in DLM (*ddn*, *fgd1*) and BDQ (*Rv0678*, *atpE*, *pepQ*) genes associated with resistance.

**Results:**

Among the 38 collected MDR-TB isolates, 7 (18.5 %) exhibited resistance to DLM, while all remained susceptible to BDQ. Analysis of the sequencing data revealed that the *ddn* gene exhibited the highest number of mutations in DLM-resistant isolates, including 18 nonsynonymous mutations and 1 indel leading to frameshift mutations. A common mutation, Gly81Ser, was present in 4 of the DLM-resistant isolates (4/7; 57.1 %). A synonymous mutation, T960C, in the *fgd1* gene was uniformly found in DLM-resistant samples. Notably, no significant mutations were observed in the *atpE*, *Rv0678*, or *pepQ* genes in any of the BDQ-susceptible isolates.

**Conclusions:**

Our study underscores the emergence of DLM resistance in a subset of MDR-TB isolates in Iran, primarily associated with mutations in the *ddn* gene. This emphasizes the ongoing necessity for TB drug resistance surveillance and research. While BDQ remains efficacious, the emergence of DLM resistance is a concerning development, warranting further exploration into resistance mechanisms and the formulation of effective TB control strategies.

## 1 Introduction

Tuberculosis (TB) remains a pressing global health issue, especially in developing countries like Iran. Given its escalating number of multidrug-resistant tuberculosis (MDR-TB) cases and extensive borders with TB-endemic nations, Iran faces heightened vulnerability to MDR-TB [1,2]. In 2018, the World Health Organization (WHO) reclassified TB drugs, positioning bedaquiline (BDQ) and delamanid (DLM) in groups A and C for MDR-TB treatment [3]. These drugs have been incorporated into the TB treatment regimens in Iran. However, to date, there has been no comprehensive study conducted on the resistance patterns of new drugs. The absence of such data underscores the urgent need to investigate resistance to newer medications, which holds major clinical and public health significance. Understanding the resistance landscape in Iran is crucial for informing effective treatment strategies and mitigating the spread of drug-resistant TB [1,4,5].

DLM is a dihydro-nitroimidazooxazole derivative activated through the mycobacterial F420 enzyme system, impacting mycolic acid synthesis [6,7]. Genes like *ddn*, *fgd1*, *fbiA*, *fbiB*, and *fbiC* influence prodrug activation and F420 biosynthesis, with mutations leading to drug resistance [[8], [9], [10], [11]].

BDQ, a diarylquinoline, disrupts mycobacterial adenosine triphosphate synthesis in replicating and dormant mycobacteria, targeting proteins encoded by *atpE*, *Rv0678*, and *pepQ* genes [[12], [13], [14]]. Mutations in these genes have the potential to confer BDQ resistance. While DLM and BDQ have shown efficacy, resistance is emerging [[15], [16], [17]].

Given the limited studies on this subject, this research assesses the susceptibility of MDR-TB strains in Iran to DLM and BDQ, while also characterizing the genomic variants associated with drug resistance.

## 2 Materials and methods

### 2.1 Study population

A total of 38 MDR-TB samples were collected consecutively, each corresponding to a unique patient, from five distinct regional tuberculosis laboratories located across various cities in Iran, including Tehran, Mashhad, Ahvaz, Shiraz, and Isfahan, between January 2018 and September 2023. Notably, all sample identification and drug susceptibility testing (DST) were conducted at the technologically advanced Tehran Regional TB Reference Laboratory, under the oversight of the Supranational Reference Laboratory, Swedish Institute for Infectious Disease Control. This state-of-the-art facility was equipped with the requisite tools for precise DST. Laboratory quality was monitored rigorously throughout the study to ensure the reliability and accuracy of our results. Quality control measures included adherence to WHO-approved methods for DST, stringent adherence to standardized protocols for molecular identification of *M. tuberculosis*, and regular calibration of laboratory equipment [18]. The study protocol received ethical approval from the Ethics Committee of the Islamic Azad University, North Tehran Branch, Tehran, Iran (IR.IAU.TNB.REC.1401.001).

### 2.2 Phenotypic identification of *M. tuberculosis*

Samples underwent staining with fluorochrome and were confirmed using Ziehl-Neelsen techniques. Subsequently, the samples were cultured on Lowenstein-Jensen medium, and growth assessments were performed on a weekly basis for up to eight weeks. The bacterial isolates were identified as *M. tuberculosis* through biochemical tests, including assessments of niacin production, nitrate reduction, and catalase activity [19,20].

### 2.3 Molecular identification of *M. tuberculosis*

Molecular identification of *M. tuberculosis* was carried out using the IS6110-based PCR assay. Genomic DNA was extracted from each specimen using the QIAamp DNA Mini Kit from QIAGEN, USA, following the provided instructions diligently. The IS6110-based PCR amplification was performed using primers designed to amplify a 123 bp segment of the IS6110 insertion element within the *M. tuberculosis* complex. Genomic DNA from *M. tuberculosis* H37Rv (ATCC 27294) and *M. fortuitum* (ATCC 49404) served as positive and negative controls, respectively [21].

### 2.4 Drug susceptibility testing

DST for Isoniazid (INH), Rifampicin (RIF), and Ethambutol (EMB) was conducted using the 1 % proportion method on Löwenstein-Jensen agar slants, in accordance with WHO guidelines [22]. DST for BDQ and DLM was performed on 7H11 Middlebrook Agar [22]. Resistance was evaluated based on the percentage of colonies exhibiting growth at critical drug concentrations: 0.2 μg/mL for INH, 40 μg/mL for RIF, 2.0 μg/mL for EMB, 0.25 μg/mL for BDQ, and 0.016 μg/mL for DLM. Pure antibiotic powder (Sigma–Aldrich, St. Louis, MO, USA), provided by Otsuka, was dissolved in DMSO. Quality control was maintained by employing the *M. tuberculosis* H37Rv strain (ATCC 27294) in DST testing.

### 2.5 PCR and sequencing

DNA extraction from clinical strains was performed using the QIAamp DNA Mini Kit and stored at −20 °C until use. Genes associated with DLM (*ddn*, *fgd1*) and BDQ (*Rv0678*, *atpE*, *pepQ*) were amplified using their respective pairs of primers [23]. The 25 μl PCR mixture was prepared with 12 μl of 2 × Taq Master Mix, 1 μl of forward and reverse primers (10 μM), 10 μl of distilled H2O, and 1 μl of genomic DNA. PCR parameters for amplification included an initial step at 95 °C for 5 min, followed by 35 cycles at 94 °C for 1 min, 58 °C for 1 min, 72 °C for 1 min, and a final extension at 72 °C for 10 min. Qualified DNA samples were sent to Microsynth Seqlab (Balgach, Switzerland) for sequencing. The sequencing reads were aligned to the *M. tuberculosis* H37Rv reference genome (NC_000962) using Bioedit (version 7.0.5.3) software.

### 2.6 Statistical analysis

Frequencies and percentages for categorical variables were calculated using MedCalc 14 statistical software.

## 3 Results

The study patients had a mean age of 50 years. Of the included patients, 63.2 % (24 out of 38) were male and 36.8 % (14 out of 38) were female. All patients had received previous treatments, with 55.3 % (21 out of 38) having undergone standard treatment (two months of INH, RIF, pyrazinamide, and EMB, followed by four months of INH and RIF) and 44.7 % (17 out of 38) receiving a fluoroquinolone-containing regimens (Table 1).

**Table 1:** Patient demographics.

Variables	Description
Mean Age	50 years old (range: 25–75 years old)
Gender	Male: 24 (63.2 %), Female: 14 (36.8 %)
Previous Treatments	Yes: 38 (100 %), No: 0 (0 %)
Treatment Regimen Category	2HRZE/4HR^a^: 21 (55.3 %),
	FQ-containing regimen^b^: 17 (44.7 %)

a Two months of isoniazid(H), rifampicin(R), pyrazinamide(Z), and ethambutol(E), followed by four months of isoniazid(H), rifampicin(R).

b Fluoroquinolone-containing regimen.

### 3.1 Drug susceptibility profiles of MDR-TB

Among the 38 MDR isolates subjected to testing, 7 isolates (18.5 %) exhibited resistance to DLM, indicating a decreased susceptibility to this specific drug. In contrast, all 38 MDR isolates demonstrated susceptibility to BDQ. Detailed results of drug susceptibility testing are provided in Table 2, Table 3.

**Table 2:** Drug susceptibility profiles of 38 MDR-TB isolates.

MDR-TB isolates	Löwenstein-Jensen Agar			7H11 Middlebrook Agar	
	RIF (40 μg/mL)	INH (0.2 μg/mL)	EMB (2.0 μg/mL)	BDQ (0.25 μg/mL)	DLM (0.016 μg/mL)
1 DRS-6	R	R	S	S	S
2 1221	R	R	R	S	R
3 2474	R	R	S	S	R
4 DRS-49	R	R	S	S	S
5219	R	R	S	S	S
6 BC-6	R	R	S	S	S
7 H-19	R	R	R	S	S
8 3935	R	R	S	S	S
9 BC-8	R	R	S	S	S
10 BC-10	R	R	S	S	S
11 219	R	R	S	S	S
12 4960	R	R	S	S	R
13 94	R	R	S	S	S
14 8922	R	R	R	S	S
15H20	R	R	S	S	R
16 5585	R	R	S	S	S
17 166	R	R	S	S	R
18 5154	R	R	S	S	S
19 3048	R	R	S	S	S
20 33	R	R	S	S	S
21 4711	R	R	S	S	S
22 9173	R	R	S	S	S
23H5	R	R	R	S	S
24 DRS-38	R	R	S	S	R
25 BC-9	R	R	S	S	S
26 3359	R	R	S	S	S
27 144	R	R	S	S	S
28 2852	R	R	S	S	S
29 1383	R	R	S	S	S
30H873	R	R	S	S	S
31 2474	R	R	S	S	S
32 M59	R	R	S	S	S
33 4711	R	R	R	S	S
34 896	R	R	S	S	S
35 840	R	R	S	S	S
36 1321	R	R	S	S	R
37 3354	R	R	S	S	S
38 720	R	R	S	S	S

RIF: Rifampicin, INH: Isoniazid, EMB: Ethambutol, DLM: Delamanid, BDQ: Bedaquiline.

**Table 3:** Delamanid and bedaquiline resistance rates.

Type of resistance	No. of MDR-TB (%) (n:38)	
	Delamanid	Bedaquiline
Resistance	7 (18.5 %)	0
Susceptible	31(81.5 %)	38(100.0)

### 3.2 Mutations associated with BDQ and DLM resistance

Successful sequencing of 38 MDR-TB strains enabled the detection of genetic mutations related to drug resistance (Table 4). In the case of DLM resistance (7 isolates), diverse mutations were observed in the *ddn* and *fgd1* genes. The mutation analysis distribution revealed 19 mutations in *ddn*, comprising 18 nonsynonymous mutations and 1 indel that led to frameshift mutations, and 11 mutations in the *fgd1* gene, involving 8 nonsynonymous mutations and 3 frameshift mutations. The most prevalent resistance mutation in the *ddn* gene, Gly81Ser, was identified in 4 out of the 7 DLM-resistant isolates (57.1 %). Notably, all DLM-resistant samples shared the T960C mutation in the *fgd1* gene, which is a synonymous mutation. One isolate exhibited an A456C mutation in the *ddn* gene, resulting in an amino acid substitution that changed a stop codon to Cysteine at position 152. Conversely, among the DLM-susceptible isolates, one showed an A to C substitution at position 119, leading to an amino acid change from Thr40 to Pro. No significant mutations were observed within the atpE, pepQ, and Rv0678 genes in the BDQ-susceptible isolates.

**Table 4:** Mutation analysis of BDQ and DLM resistance genes in 38 MDR-TB isolates.

Drugs	Susceptible/resistance	Resistant Genes	Nucleotide Substitution	Amino acid change	Number of isolates
**Delamanid**	Susceptible isolates	*ddn*	A119C	Thr40Pro	1
		*Fgd1*	NC	NC	0
	Resistant isolates	*ddn*	A151C	Thr51Pro	2
			C192T	lle64Phe	1
			G241A	Gly81Ser	4
			G229C	Ala77Pro	1
			A266C	Tyr89Ser	1
			A272C	Asp108Ala	1
			A328C	Thr110Pro	1
			A343C	Thr115Pro	1
			C348G	Asp116Glu	1
			A418C	Thr140Pro	2
			A422C	Asp141Ala	1
			C447G	Cys149Trp	1
			A456C	stop152Cys	1
			Position 92 Del-G^a^	Fs31^a^	1
		*Fgd1*	T750G	Phe250Cys	1
			G806A	Arg269Cys	2
			C819A	Gly273Arg	2
			G839A	Arg 280Cys	1
			Position 883 Ins-A	Fs94	1
			T932A	Val310Asp	1
			T960C	NC	7
			Position 962 Del-CG	320Fs	1
			T965C	Phe322Ser	1
			Position 46 Ins-A	Fs16	1
**Bedaquiline**	Susceptible isolates	*Rv0678*	NC	NC	0
		*atpE*	NC	NC	0
		*pepQ*	NC	NC	0
	Resistance isolates	*Rv0678*	NC	NC	0
		*atpE*	NC	NC	0
		*pepQ*	NC	NC	0

a Del: deletion, Fs: frameshift, Ins: insertion, NC: no change.

## 4 Discussion

The results of this study offer valuable insights into the drug susceptibility profiles and genetic mutations associated with resistance in MDR-TB isolates, which play a pivotal role in the development of effective treatment strategies and the prevention of the further spread of drug-resistant TB.

In our investigation, it was notable that 18.5 % of the 38 MDR-TB isolates exhibited resistance to DLM, an essential drug in MDR-TB treatment. This diminished susceptibility raises concerns. Within the DLM-resistant samples, we observed a range of mutations within the ddn and *fgd1* genes, encompassing both nonsynonymous mutations and frameshift mutations, which can significantly influence the functionality of these genes. The prevailing resistance mutation in the *ddn* gene, Gly81Ser, was prevalent in the majority of DLM-resistant isolates. Moreover, a unique mutation, A456C, was identified in the ddn gene, resulting in an amino acid substitution from a stop codon to Cysteine at position 152 in one isolate. These findings emphasize the intricate and diverse nature of genetic mutations linked to DLM resistance, underscoring the necessity for personalized treatment strategies tailored to MDR-TB patients [[24], [25], [26], [27]].

Notably, in previous studies, a varying percentage of MDR-TB isolates (ranging from 5 % to 30 %) displayed resistance to DLM, which is consistent with our findings [23,28]. Additionally, polymorphisms within genes like *ddn* and *fgd1* have been associated with in vitro DLM resistance [23,28]. The *ddn* gene, responsible for encoding an F420-dependent mycobacterial nitroreductase crucial for prodrug activation, plays a pivotal role in DLM resistance [29]. Loss of this activation ability leads to resistance to DLM. Similarly, in a study by Liu et al. nonsynonymous mutations in the ddn gene were found in MDR-TB isolates, affirming our results [30]. Furthermore, in alignment with prior research, several mutations were identified in the fgd1 gene, potentially contributing to increased DLM resistance [28,31].

In contrast, unlike DLM, our study revealed the complete susceptibility of all 38 MDR isolates to BDQ, without any detected genetic mutations within the studied genes. Consistently, previous studies have also consistently reported high susceptibility rates for MDR-TB strains to BDQ, surpassing 95 % [23]. Furthermore, both our study and prior research did not identify any *atpE* mutations associated with BDQ resistance, suggesting that alternative mechanisms may underlie BDQ resistance [23].

In Iran, the treatment of MDR-TB typically involves individualized regimens tailored to the patient's specific drug susceptibility profile. The standard treatment approach often includes a combination of second-line anti-TB drugs, such as fluoroquinolones. The introduction of newer drugs like DLM and BDQ has expanded the therapeutic options for MDR-TB, particularly for cases resistant to conventional medications. However, despite the availability of these newer drugs, challenges persist in optimizing treatment outcomes and preventing the development of resistance. Factors such as medication adherence, drug interactions, and the presence of comorbidities could influence treatment success rates and the emergence of resistance.

Also, our study offers an initial but valuable insight into this area; the significance of conducting a national survey on resistance to new drugs in Iran cannot be overstated. Such a survey would yield critical insights into the current epidemiology of resistance patterns, essential for guiding regional and global TB control efforts. Iran's substantial role within the WHO Eastern Mediterranean Region underscores the necessity of monitoring and addressing drug resistance within the country. Insights gleaned from a national survey in Iran would not only inform local TB control strategies but also bolster collaborative efforts aimed at combating drug-resistant tuberculosis in neighboring countries and the broader region. With the emergence of BDQ resistance observed in Pakistan, there is an urgent need for proactive measures to monitor and address drug resistance trends within Iran [[32], [33], [34]]. Conducting a larger-scale survey encompassing a broader range of MDR-TB isolates from various regions of Iran would furnish a more comprehensive understanding of resistance patterns and trends over time. This, in turn, would facilitate informed decision-making regarding TB treatment and control strategies, ultimately contributing to endeavors aimed at mitigating the spread of drug-resistant TB.

The study has some limitations that should be taken into consideration. Firstly, the sample size, comprising 38 MDR-TB isolates, is relatively small. While informative, it may not fully represent the prevalence of drug resistance in the country and the diversity of genetic mutations associated with drug resistance. Second, regional variation is a concern, as the findings may be influenced by geographic differences in MDR-TB strains and their resistance patterns. Third, while the study identifies the absence of significant mutations in BDQ-susceptible isolates, the potential for cross-resistance or the emergence of new mutations over time may not be entirely captured in this cross-sectional analysis. Finally, while our study successfully identifies mutations associated with DLM resistance, we did not thoroughly explore other factors such as patient demographics, previous treatment history, co-infections, endemicity, HIV status, and poor housing/sanitation which could also impact drug resistance development. This limitation highlights the need for future research efforts to adopt a more comprehensive approach, considering a wider range of variables that could influence drug resistance mechanisms.

In conclusion, this study's findings shed light on the complex landscape of DLM resistance and BDQ susceptibility among MDR-TB isolates. The identification of DLM-resistant strains emphasizes the need for vigilance in treatment selection and underscores the importance of personalized therapeutic strategies. On the other hand, the universal BDQ susceptibility is an encouraging sign for clinicians seeking effective treatment options. Nevertheless, the study emphasizes the ongoing requirement for research and a cautious approach to integrating genetic findings into clinical practice, ensuring their meaningful impact on MDR-TB management.

## CRediT authorship contribution statement

**AmirHossein Akbari Aghababa:** Writing – original draft, Methodology, Investigation. **Mohammad Javad Nasiri:** Writing – review & editing, Writing – original draft, Validation, Supervision, Investigation, Conceptualization. **Parviz Pakzad:** Data curation, Conceptualization. **Elnaz Sadat Mirsamadi:** Methodology.

## Declaration of competing interest

The authors declare that they have no known competing financial interests or personal relationships that could have appeared to influence the work reported in this paper.
